# Sex-Related Specificities of Sensory Profiles in Autism Spectrum Disorder: Impact on Clinical Functioning

**DOI:** 10.3390/medicina62101909

**Published:** 2026-10-02

**Authors:** Mariangela Pezone, Raffaele Garotti, Elma Sgueglia, Rosamaria Siracusano, Valeria Maffettone, Chiara Avitabile, Adele Maisto, Carmela Bravaccio, Maria Pia Riccio

**Affiliations:** 1 Residency Program of Child and Adolescent Neuropsichiatry, Department of Medical and Translational Sciences, Federico II University, 80131 Naples, Italy; 2 Child and Adolescent Neuropsychiatry, Maternal-Infant Department, Federico II University Hospital, 80131 Naples, Italy; 3 Department of Mental and Physical Health and Preventive Medicine, University of Campania “Luigi Vanvitelli”, 80131 Naples, Italy; 4 Department of Psychology, University of Campania “Luigi Vanvitelli”, 80131 Naples, Italy; 5 Department of Medical and Translational Sciences, Child and Adolescent Neuropsychiatry, Federico II University, 80131 Naples, Italy

**Keywords:** Autism Spectrum Disorder, sensory profile, sex differences

## Abstract

*Background and Objectives:* Sensory anomalies are a distinctive feature of Autism Spectrum Disorder (ASD) individuals and appear to differ in intensity, frequency, and subtype between male and female subjects. According to the diagnostic framework of the DSM-5-TR, these sensory patterns are key clinical indicators. The objectives of this study were to investigate sensoriality in ASD, to compare sensory profiles in ASD subjects of both sexes and evaluate potential correlations with clinical profiles. *Materials and Methods:* A total of 94 ASD participants were enrolled at the Children and Adolescents Neuropsychiatry Department of the University of Naples Federico II. The population was assessed through psychometric (cognitive and adaptive levels, and intensity of autism symptoms) and sensory characterization (using the Short Sensory Profile questionnaire). Subsequently, a Mann–Whitney U test was performed to evaluate differences in sensory profiles between males and females. Spearman’s correlation was used to assess significant associations between the sensory profile and cognitive level, adaptive level, and ASD symptom intensity. Subsequently, for the correlations that emerged as significant, an analysis was performed using a General Linear Model (GLM), including age and Intelligence Quotient (IQ) as covariates in the model. *Results:* No statistically significant differences in the sensory profile emerged between male and female subjects. Sensory anomalies do not appear to be correlated with cognitive level. Although some statistically significant correlations emerged between the sensory profile, the AQ, and the ADOS-2 comparison score, these results were not confirmed after a GLM analysis was performed using the participants’ age and IQ as covariates. *Conclusions:* The observed results appear partially in line with the existing literature. Following the GLM analysis, it was further observed that variables such as age and IQ play a crucial role in interpreting the functional profile of individuals with ASD. At present, the study has several limitations (the exclusive use of parent-report instruments, an observational design, the lack of a control group) which could affect the robustness of the observed findings. Therefore, further studies are deemed necessary to better define the sex-specific sensory characteristics of ASD individuals.

## 1. Introduction

Atypical sensory profiles are a highly prevalent feature in Autism Spectrum Disorder (ASD) individuals. In fact, one of the diagnostic criteria currently set forth by the Diagnostic and Statistical Manual of Mental Disorders, Fifth Edition, Text Revision (DSM-5-TR)—categorized under the B criteria, including repetitive and stereotyped behaviors—describes symptoms as: “Hyper- or hypo-reactivity to sensory input or unusual interest in sensory aspects of the environment” [1]. Globally, many studies focused on sensory anomalies in ASD individuals to contribute to a clearer neurobiological and phenotypic definition. Sensory anomalies can affect any sensory channel (sight, hearing, touch, etc.) and may be associated with an individual’s unique behaviors and response thresholds. For instance, referring to the theoretical framework proposed by Dunn W., we can identify four sensory processing patterns (derived from the combination of response thresholds, behavioral self-regulation strategies, and the interaction between these two factors): sensory seeking, sensory avoiding, sensory sensitivity, and low registration [2]. These aspects impact the functioning of ASD individuals, contributing to the impairment of their ability to adapt to different contexts and exacerbating any concomitant dysfunctional behaviors [3]. Indeed, it is well known that sensory abnormalities can also affect the social skills of ASD individuals, leading for example to difficulties in integrating social messages provided by other people; such aspects could be underlying by abnormalities in brain circuits that regulate both aspects of sociality as well as sensory processing, such as, for example, the anterior cingulate cortex, the superior temporal cortex, and the insula [4]. Furthermore, the scientific literature is increasingly exploring differences in symptomatic expression based on sex. Specifically, the ASD phenotype in females may differ both in the intensity of the symptoms expressed and in the potential comorbidities that may develop over time [5]. In fact, for example, females with ASD tend to present a higher prevalence of internalizing comorbidities (such as depression and anxiety), which often serve as the primary reason for medical consultation and can lead to a missed ASD diagnosis [6]. For instance, it is well known that ASD women tend to employ camouflage strategies to a greater extent in social contexts; these behaviors result in higher levels of perceived social reciprocity, which can mask the underlying ASD symptomatology [7]. Sensory processing anomalies are also being studied to evaluate potential differential characteristics based on sex. For instance, Osorio J.M.A. et al. assessed how, in a sample of ASD youth, females exhibit greater differences in the domains of proprioceptive and auditory sensitivity [8]. Differences in sensory processing are also present from early childhood: Harris H.K. et al. highlighted that, in a sample of ASD preschool-aged children, females tend to exhibit more sensory seeking behaviors compared to their male peers [9]. In light of the above, the purpose of the present study is to characterize a cohort of ASD individuals and compare sensory profiles between male and female subjects. A secondary objective is to evaluate the potential impact of the sensory profile on the clinical phenotype of both male and female subjects, comparing any observed differences.

## 2. Materials and Methods

### 2.1. Study Population

This observational-retrospective study was conducted at the Interdepartmental Program of Child Neuropsychiatry at the University of Naples Federico II. The study included 94 participants consecutively referred for clinical observation due to suspected ASD or for psychodiagnostic re-evaluation of an existing ASD diagnosis between 2022 and 2024. Inclusion criteria were: an ASD diagnosis consistent with the DSM-5 and DSM-5-TR; age between 3 and 14 years; absence of ongoing psychopharmacological treatment; stability of rehabilitative treatment for at least 4 months; and signed informed consent from a parent or legal guardian. Individuals who did not meet all the aforementioned criteria were excluded. Furthermore, subjects with syndromic ASD (e.g., Fragile X Syndrome) and those with internal medical conditions that could alter the assessed parameters (such as comorbid epilepsy or other neurological disorders) were also excluded.

### 2.2. Psychometric Evaluation

For the enrolled subjects, clinical characterization was performed through cognitive or developmental tests (Wechsler Intelligence Scale for Children-Fourth Edition, Leiter-3) [10,11], adaptive profile assessment (Vineland Adaptive Behavior Scales, Second Edition) [12], and the administration of the Autism Diagnostic Observation Schedule, Second Edition (ADOS-2) to evaluate the intensity of autistic symptom expression [13]. The Intelligence Quotient (IQ) was extracted as data for our analysis. For the adaptive assessment, instead, the Adaptive Quotient (AQ) was extracted from the Vineland Adaptive Behavior Scales, Second Edition interview. Finally, the comparison score, indicating the intensity of the autistic symptomatology, obtained by the ADOS-2 test was extracted. To characterize the sensory profile, the Short Sensory Profile (SSP) questionnaire was administered [14]. This tool consists of 38 items evaluating different sensory domains and is structured into eight scales: seven subscales divided by sensory channel (‘Tactile Sensitivity’, ‘Taste/Smell Sensitivity’, ‘Movement Sensitivity’, ‘Auditory Filtering’, ‘Low Energy/Weakness’, ‘Visual/Auditory Sensitivity’) and behavioral response type (‘Hypo-responsivity/Sensation Seeking’), plus a total score. The SSP allows for an assessment of how much the sensory profile deviates from typical development, categorized as ‘Typical Performance’, ‘Probable Difference’, or ‘Definite Difference’. This tool is validated for parent/caregiver self-administration for children up to 14 years of age. The data obtained were analyzed to assess potential differences in the sensory profile between males and females in the study sample.

### 2.3. Statistical Analysis

Regarding statistical analysis, a descriptive characterization of the study population was first performed for both demographic and clinical variables. The distribution of study variables was assessed using the Shapiro–Wilk test, which indicated a non-normal distribution for several variables (*p* < 0.05). Subsequently, the sample was divided by sex (male/female): a comparison statistic using the Mann–Whitney U test for independent samples was set up to evaluate significant differences in SSP scores between the two subgroups. Significant correlations were then assessed (via Spearman’s bivariate analysis) between the sensory profile and clinical variables (cognitive and adaptive levels, autistic symptom intensity). Specifically, bivariate correlation analyses were conducted between SSP scores and Intelligence Quotient (IQ), Adaptive Quotient (AQ), and the ADOS-2 comparison score. Results with a *p*-value < 0.05 were considered statistically significant. All statistical analyses were performed anonymously using Statistical Package for the Social Sciences (SPSS) Version 29.

### 2.4. Ethical Considerations

The study was conducted in accordance with the Declaration of Helsinki and approved by the Ethics Committee ‘Campania 3’ (Protocol No. 78/2024, 26 March 2024). For all subjects, informed consent was signed by the parents/caregivers before the start of the diagnostic assessments at our facility. This informed consent also provided availability for the anonymous use of the subjects’ data for research purposes.

## 3. Results

The study sample consisted of 94 subjects, of whom 32 were female and 62 were male (34% F; 66% M). Based on the severity levels estimated according to the criteria of the DSM-5 and DSM-5-TR, the majority of the subjects presented with severity levels 1 and 2. The IQ and AQ parameters were similar between the two groups, as was the comparison score for the ADOS-2 test. The administration of cognitive tests was not possible for subjects aged less than 5 years or due to the limited cooperation provided: this characterization was only possible to perform for 51 subjects. Please refer to Table 1 for a complete and detailed demographic characterization of the sample.

The Mann–Whitney U test for independent samples, aimed at evaluating differences in the sensory profile via SSP scores between male and female subjects, yielded no statistically significant results (see Table 2). Indeed, the scores obtained by both groups on both the individual subscales and the total scale were comparable.

Despite the lack of observed differences, a Chi-square analysis was performed to confirm the data by comparing the distribution of SSP total score categories between males and females; this analysis also yielded no statistically significant results (chi-square 2.01, *p* 0.366). Bivariate correlation analysis revealed two statistically significant inverse correlations within the female group. These involved the scores of the ‘Auditory Filtering’ subscale (Spearman Rho = −0.542, *p* = 0.008, 95% CI [−0.785, −0.155]) and the ‘Hyporesponsiveness/Sensation Seeking’ subscale (Spearman Rho = −0.551, *p* = 0.006, 95% CI [−0.790, −0.168]). Based on the Rho coefficients, both correlations were moderate-to-strong. No statistically significant correlations between SSP scores and the ADOS-2 comparison score were found within the male group. For the 51 subjects (30 M; 21 F) for whom intellectual assessment results were available, a bivariate analysis between SSP and IQ scores was conducted, which revealed no statistically significant results. A statistically significant direct correlation was found between “Tactile Sensitivity” and AQ (Spearman Rho 0.379, *p*-value 0.017, CI [0.063, 0.626]). A moderate effect is observed for this correlation. In the female group, two statistically significant direct correlations were observed between the AQ score and both the ‘Hyporesponsiveness/Sensation Seeking’ (Spearman Rho = 0.479, *p* = 0.015, 95% CI [0.091, 0.741]) and ‘Auditory Filtering’ (Spearman Rho = 0.405, *p* = 0.044, 95% CI [0.010, 0.696]) subscales. In both cases, the effect size was moderate. All these results are summarized in Table 3 and Table 4.

Given the age heterogeneity within the study sample and the potential influence of IQ on both the adaptive profile and the ADOS-2 comparison score, an additional analysis was conducted using a General Linear Model (GLM). In this analysis, age and IQ were entered as covariates to evaluate the impact of these variables on the observed results. Due to the presence of non-normally distributed values, and to ensure the robustness of the analysis, this was integrated with a bootstrap procedure. For the “Tactile Sensitivity” subscale, the GLM applied to the dependent variable AQ showed an excellent overall fit (R^2^ = 0.443, F(5, 37) = 5.885, *p* < 0.001). Among the variables included in the model, IQ was found to be the only highly significant predictor (F(1, 37) = 23.729, *p* < 0.001), showing a particularly large effect size (partial eta squared = 0.391). Conversely, no significant effects emerged for the age covariate (*p* = 0.945), for sex (*p* = 0.677), or for the interaction between sex and the “Tactile Sensitivity” subscale (*p* = 0.652). For the subscale for “Hyporesponsiveness/Sensation Seeking”, the GLM applied also showed an excellent overall fit (R^2^ = 0.464, F(5, 37) = 6.401, *p* < 0.001). Among the variables included in the model, IQ was found again to be the only highly significant predictor (F(1, 37) = 17.007, *p* < 0.001), showing a particularly large effect size (partial eta squared = 0.315). Conversely, no significant effects emerged for the age covariate (*p* = 0.721), for sex (*p* = 0.423), or for the interaction between sex and the “Hyporesponsiveness/Sensation Seeking” subscale (*p* = 0.457). Finally, for the “Auditory Filtering” subscale, the GLM applied showed an excellent overall fit (R^2^ = 0.463, F(5, 37) = 6.389, *p* < 0.001 ). Among the variables included in the model, IQ was found to be the only highly significant predictor (F(1, 37) = 17.291, *p* < 0.001), showing a particularly large effect size (partial eta squared = 0.318). Conversely, no significant effects emerged for the age covariate (*p* = 0.569), for sex (*p* = 0.755), or for the interaction between sex and the “Auditory Filtering” subscale (*p* = 0.790). In conclusion, therefore, sex does not appear to act as a moderator on the AQ score; instead, a large portion of the observed effect is attributable to IQ. Furthermore, for the “Auditory Filtering” subscale, the GLM applied to the dependent variable ADOS-2 comparison score showed a statistically significant overall fit (R^2^ = 0.316, F(5, 32) = 2.956, *p* = 0.026). Among the variables included in the model, no single predictor reached statistical significance; the effect of IQ trended toward significance but did not cross the threshold (F(1, 32) = 3.463, *p* = 0.072, partial eta squared = 0.098). Similarly, no significant effects emerged for the age covariate (*p* = 0.543), for sex (*p* = 0.281), or for the interaction between sex and the “Auditory Filtering” subscale (*p* = 0.282). Finally, the GLM applied for the “Hyporesponsiveness/Sensation Seeking” subscale showed a statistically significant overall fit (R^2^ = 0.324, F(5, 32) = 3.061, *p* = 0.023). Among the variables included in the model, IQ was found to be the only statistically significant predictor (F(1, 32) = 4.992, *p* = 0.033), showing a moderate-to-large effect size (partial eta squared = 0.135). Conversely, no significant main effects emerged for the age covariate (*p* = 0.302), for sex (*p* = 0.177), or for the interaction between sex and the “Hyporesponsiveness/Sensation Seeking” subscale (*p* = 0.155). Therefore, the highlighted statistically significant correlations could be attributed to the lack of control over certain variables, such as age and IQ. When considering these factors, sex does not appear to have a moderating role on either the adaptive profile or autistic symptom severity.

## 4. Discussion

The evaluation of our sample revealed no significant differences when comparing the sensory profiles of males and females using U-Mann–Whitney analysis. These findings appear to be partially in line with the available literature on the subject. Specifically, Bitsika V. et al. observed no significant discrepancies in the sensory profiles of their sample of ASD individuals when comparing them on the basis of biological sex [15]. Conversely, other studies have observed both quantitative and qualitative differences in sensory profiles, with greater symptom intensity in ASD female subjects, particularly regarding auditory and kinesthetic sensitivity [8,9]. Beyond phenotypic aspects, there appear to be underlying neurobiological differences influencing the distinct sensory behaviors of ASD males and females. For instance, research has shown that differing patterns of neural connectivity, particularly within the frontal cortex, may correlate with sensory hyper-responsivity in ASD females, a finding not observed in males [8]. Our results also indicate the absence of a significant correlation between sensory profiles and IQ scores in either subgroup. This finding is largely consistent with the existing literature, which suggests that IQ does not significantly impact sensory processing [16]. Despite this, the sources are not in complete agreement on this matter: indeed, Narzisi A. et al. highlight how there may be a differing expressivity of sensory anomalies between low- and ASD high-functioning individuals, particularly concerning the auditory and proprioceptive domains [17].

Conversely, within our sample, certain sensory aspects (specifically, the ‘Hyporesponsiveness/Sensation Seeking’ and ‘Auditory Filtering’ subscales) were found to correlate with the ADOS-2 comparison score; however, this correlation was observed exclusively in the female group and not in the male group. This finding aligns relatively well with previous observations in the literature [18]. However, after applying GLM statistics, sex was no longer found to be associated with the scores obtained on either of these scales when controlling for age and IQ. Therefore, this result should be interpreted in light of the strong impact that IQ can exert on the autistic phenotype. Indeed, it is well known that IQ can influence the expression of autistic symptomatology, whereby a lower IQ inflates the scores detectable in psychometric assessments, highlighting a more severe clinical phenotype [19]. Regarding the adaptive profile, significant correlations were observed in both groups, albeit for different sensory aspects. Overall, the direction of the correlations indicates that poorer sensory performance may be associated with a worse adaptive outcome. For the female group, this association relates to the ‘Hyporesponsiveness/Sensation Seeking’ and ‘Auditory Filtering’ subscales, whereas in the male group, it relates to the ‘Tactile Sensitivity’ and ‘Hyporesponsiveness/Sensation Seeking’ subscales.

This finding could align with the literature: Neufeld J. et al. observed in an ASD cohort that sensory seeking and hypersensitivity behaviors negatively correlate with personal autonomy, thereby impacting the overall functioning of ASD individuals [20]. Kadlaskar G. et al. also highlighted how the sensory profile negatively impacts not only adaptive functioning but also socio-communicative skills and symptoms of inattention and hyperactivity; furthermore, this impact was found to be more pronounced in individuals presenting with more severe sensory anomalies [21]. Among the observed results, the findings regarding ‘Tactile Sensitivity’ in the male group are of particular interest. The existing literature suggests that tactile abnormalities do not uniformly correspond to a decline in clinical profiles. Foss-Feig et al. observed that tactile hyposensitivity and sensation-seeking behaviors were linked to poorer social and communication skills, whereas tactile hypersensitivity exerted no significant impact on the overall clinical profile [22]. Similarly, other researchers reported no significant association between tactile sensory issues and adaptive functioning, despite clear evidence linking these abnormalities to behavioral difficulties and psychopathological symptoms, such as anxiety [23]. Nevertheless, the majority of evidence converges on the fact that sensory profiles—including tactile sensitivity— affect adaptive functioning, as also previously shown by Rogers et al. in a clinical sample using psychometric instruments similar to the reported ones [24]. In relation to sex, Asaridou et al. observed that while differences in the tactile profile exist between males and females, these did not prove to be autism-specific [25]. Regarding the results highlighted in relation to the AQ, no further significant effects were observed after a GLM analysis was conducted using age and IQ as covariates. Specifically, it was found that most of the variance in the AQ score is primarily attributable to the IQ score. This aspect is documented in the literature and is therefore consistent with our observations [26]. Age may also play an important role in sensory abnormalities and in the developmental trajectory of individuals with ASD. Indeed, it is well known that certain aspects related to sensory abnormalities (such as sensory hyperreactivity) may improve with age [27].

### Strengths and Limitations

The present study aimed to evaluate potential differences in sensory profiles and their impact on functioning within a cohort of ASD participants, stratified by sex. The current literature findings on this topic remain inconsistent; our study aligns with previous observations that reported no significant sex-based differences. One of the strengths of this study is the application of stringent inclusion and exclusion criteria during recruitment. Indeed, it is well known that various clinical conditions, including other neurodevelopmental disorders, can co-occur with ASD [28,29]. These conditions could have altered the observed results and distorted the conclusions, as they have the potential to modify the participants’ clinical parameters.

This study has several limitations: the sample size of enrolled subjects is relatively small, which may have affected the robustness of the analyses and limited the inferential power of our results. Furthermore, our sample exhibited an imbalanced male-to-female ratio. This asymmetry may be attributed to the epidemiology of ASD, as males are known to be more frequently affected, with sex ratios varying across studies but averaging approximately 3:1 [30]. Indeed, it is well known that various factors (genetic, hormonal, etc.) can contribute to this differential distribution of ASD based on sex [31]. Furthermore, the cross-sectional design of this study precludes the determination of causal relationships from the analyses performed. Additionally, the lack of a control group represents a limitation that could have otherwise enhanced the robustness of the findings. Furthermore, the analyses did not evaluate all possible psychometric indices; specifically, only the total IQ and AQ scores were selected for correlation analyses, excluding additional indices derivable from the tests (such as cognitive ability indices and specific adaptive functioning sub-domains). A further limitation of our study could also concern the use of a parent-report instrument for the exploration of sensory abnormalities. Future studies should, therefore, involve larger samples and include a broader range of psychometric indices. The ultimate goal is to define care pathways and management strategies that are more tailored to the individual patient.

## 5. Conclusions

In conclusion, our observational study aimed to evaluate potential differences in the sensory profile within a cohort of ASD subjects divided on the basis of sex. By statistical analysis, no significant differences were appreciated between males and females in any of the sensory areas explored by the SSP questionnaire.

Although some statistically significant correlations emerged between the sensory profile, the AQ, and the ADOS-2 comparison score, these results were not confirmed after a GLM analysis was performed using the participants’ age and IQ as covariates.

The observational nature of our study, as well as the small sample size, currently limit the generalizability of the observed results as well as the possibility of defining a causal link relative to the observed correlation. Furthermore, the use of a parent report instrument for the exploration of sensory abnormalities could limit the robustness of our results. For these aspects, further studies are considered fundamental to study sensory abnormalities in ASD, also allowing for a differential characterization on the basis of sex, and evaluating their impact on global functioning.

## Figures and Tables

**Table 1:** Demographic and clinical characteristics of the study population.

Age (year)	6.98 (2.38) *
Sex	
Males	62 (66) **
Females	32 (34) **
Severity level	
Level 1	38 (40.4) **
Level 2	37 (39.4) **
Level 3	19 (20.2) **
Total Score SSP (male sex)	
Typical performance	23 (37.1) **
Probable difference	14 (22.5) **
Definite difference	25 (40.3) **
Total Score SSP (female sex)	
Typical performance	16 (50) **
Probable difference	4 (12.5) **
Definite difference	12 (37.5) **
AQ	64.5 (22.07) *
Males	62.79 (19.8) *
Females	67.16 (25.41) *
IQ	82.65 (19.05) *^,§^
Males	79.97 (16.97) *
Females	86.48 (21.53) *
ADOS-2 comparison score	6.86 (1.91)
Males	7.06 (1.85) *
Females	6.56 (2.01) *

* Values expressed as mean (standard deviation); ** values expressed as *n* (percentage in the sample); ^§^ value obtained only from 51 subjects in the sample. SSP: Short Sensory Profile; AQ: Adaptive Quotient; IQ: Intelligence Quotient.

**Table 2:** Results of the Mann–Whitney U analysis for independent samples. Values for the first two columns are expressed as the median. No significant results were observed. In the *p*-value column, the effect size (expressed as Rosenthal’s r coefficient) is reported in parentheses.

	Males	Females	U	*p*-Value
Tactile sensitivity	30	31	984	0.95 (0.006)
Taste/smell sensitivity	15	14	960	0.8 (0.02)
Movement sensitivity	14	14	908	0.488 (0.07)
Underresponsive/seeks sensation	22	27	1116	0.322 (0.1)
Auditory filtering	21	23	1014	0.86 (0.02)
Low energy/weakness	27	30	1062	0.564 (0.06)
Visual/auditory sensitivity	20.5	21	958	0.785 (0.03)
Total	146	164	1065	0.557 (0.06)

**Table 3:** Spearman bivariate analysis (male group) between SSP questionnaire scores and Intelligence Quotient (IQ), Adaptive Quotient (AQ), and ADOS-2 comparison score for male patients. * Statistically significant values (*p* < 0.05).

Spearman’s Rho	IQ	AQ	Comparison Score (ADOS)
SSP—Taste and smell sensitivity	−0.138	0.294	−0.202
SSP—Movement sensitivity	−0.061	0.036	−0.110
SSP—Hyporesponsiveness/sensation seeking	0.230	0.318 *	−0.141
SSP—Auditory filtering	0.317	0.251	−0.239
SSP—Low energy/weakness	−0.246	0.007	0.042
SSP—Tactile sensitivity	0.339	0.379 *	−0.177
SSP—Visual/auditory sensitivity	0.074	0.176	0.006
SSP—Total	−0.038	0.201	−0.089

**Table 4:** Spearman bivariate analysis (female group) between SSP questionnaire scores and Intelligence Quotient (IQ), Adaptive Quotient (AQ), and ADOS-2 comparison score for female patients.

Spearman’s Rho	IQ	AQ	Comparison Score (ADOS)
SSP—Tactile sensitivity	0.133	0.159	−0.306
SSP—Taste and smell sensitivity	−0.079	0.229	−0.035
SSP—Movement sensitivity	0.03	−0.033	−0.161
SSP—Hyporesponsiveness/sensation seeking	0.156	0.479 *	−0.551 *
SSP—Auditory filtering	0.259	0.405 *	−0.542 *
SSP—Low energy/weakness	0.043	0.148	−0.137
SSP—Visual/auditory sensitivity	0.306	0.083	−0.257
SSP—Total	0.195	0.314	−0.379

* Statistically significant values (*p* < 0.05).

## Data Availability

The data used for the study are available upon reasonable request to the corresponding author.
